# Body Mass Index at Pediatric Leukemia Diagnosis and the Risks of Relapse and Mortality: Findings from a Single Institution and Meta-analysis

**DOI:** 10.1155/2018/7048078

**Published:** 2018-11-01

**Authors:** Ashleigh M. Saenz, Stacie Stapleton, Raquel G. Hernandez, Greg A. Hale, Neil A. Goldenberg, Skai Schwartz, Ernest K. Amankwah

**Affiliations:** ^1^Department of Epidemiology and Biostatistics, College of Public Health, University of South Florida, Tampa, FL, USA; ^2^Department of Pediatrics, Johns Hopkins University School of Medicine, Baltimore, MD, USA; ^3^Cancer and Blood Disorders Institute, Johns Hopkins All Children's Hospital, St. Petersburg, FL, USA; ^4^Office of Medical Education, Johns Hopkins All Children's Hospital, St. Petersburg, FL, USA; ^5^Department of Oncology, Johns Hopkins University School of Medicine, Baltimore, MD, USA; ^6^Clinical and Translational Research Organization, All Children's Research Institute, Johns Hopkins All Children's Hospital, St. Petersburg, FL, USA

## Abstract

High body mass index (BMI) is associated with relapse of certain adult cancers, but limited knowledge exists on its association with pediatric leukemia relapse. We evaluated the association between overweight/obesity (BMI ≥ 85^th^ percentile) at pediatric leukemia diagnosis and relapse or mortality. A meta-analysis combining our findings with those of previous studies was also performed. The study included 181 pediatric leukemia patients. Sporadic missing data were multiply imputed, and hazard ratios (HR) and 95% confidence intervals (95% CI) were calculated using Cox proportional hazard. Age- and sex-adjusted analysis for patients ≥10 years showed a trend towards increased risk of relapse for overweight/obese patients (HR = 2.89, 95% CI = 0.89–9.36, *p*=0.08) that was not evident among children<10 years (HR = 0.52, 95% CI = 0.08–3.54, *p*=0.49). We observed a statistically significant association between mortality and obesity status in unadjusted models (imputed: HR = 2.54, 95% CI = 1.15–5.60, *p*=0.021; complete set: HR = 2.72, 95% CI = 1.26–5.91, *p*=0.011) that was not statistically significant in both age- and sex-adjusted and multivariable adjusted analyses. The pooled estimate of our finding and previous studies showed an association between overweight/obese and increased risk of mortality for ALL (HR = 1.39, 95% CI = 1.16–1.46) and AML (HR = 1.64, 95% CI = 1.32–2.04). Although our study did not observe statistically significant associations due to a small sample size, the meta-analyses revealed an increased risk of mortality for overweight/obese patients. The findings of our study suggest an association of obesity status with relapse in children ≥10 years. However, our study was based on a small sample size from a single institution, and this association needs to be investigated in larger, multicenter studies.

## 1. Introduction

Leukemia is the most prevalent pediatric malignancy and the leading cause of cancer-related death among youth [1]. Acute lymphocytic leukemia (ALL), the most common form of leukemia, independently represents more than a quarter of all cancer diagnoses among children aged 0–14 years [2, 3]. Substantial improvement in therapy has resulted in a dramatic increase in the survival rate for leukemia over the past five decades, with 5-year overall survival now at about 80% for ALL [2, 3] and 60% for acute myeloid leukemia (AML) [4, 5]. However, approximately 20–40% of pediatric leukemia patients develop relapse and become refractory to treatment [6–9].

Obesity has been associated with relapse of certain adult cancers [10–12], but limited knowledge exists on its association with pediatric leukemia relapse. Furthermore, findings from the few studies in pediatrics have been inconsistent with some studies reporting an increased risk of relapse with obesity [13–15] while others have reported no association [16–21].

The purpose of this study was to evaluate the association between body mass index (BMI) at diagnosis and pediatric (<21 years) leukemia relapse by comparing the risk for non-overweight to overweight/obese patients. We hypothesized that pediatric leukemia patients with a higher BMI at diagnosis are more likely to relapse than pediatric leukemia patients with a normal or lower BMI at diagnosis. We also evaluated the association between BMI at diagnosis and overall mortality.

## 2. Materials and Methods

### 2.1. Study Design and Study Population

We conducted a retrospective analysis on children diagnosed with leukemia at Johns Hopkins All Children's Hospital (JHACH, St. Petersburg, FL). JHACH is a tertiary children's hospital with an established Cancer and Blood Disorders Institute that serves as the largest pediatric cancer center in West Florida. The study was approved by JHACH Institutional Review Board, with waiver of informed consent granted.

**Figure 1:**
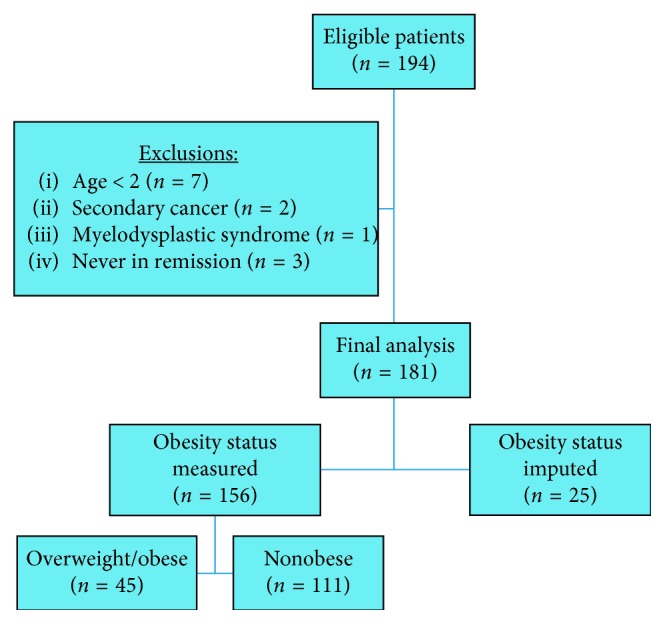
Diagram of eligible and included leukemia patients.

All patients with leukemia treated at JHACH were considered eligible for the study. However, the analysis was limited to patients diagnosed between April 2005 and December 2014 and followed through June 2016, because BMI was not consistently collected in our database before this period. Participants were identified via an electronic health record-derived data warehouse using the first recorded International Classification of Diseases, Ninth Revision (ICD-9) codes 204.00–205.92. Diagnoses included ALL, AML, and chronic myeloid leukemia (CML). We reviewed the records of 194 patients with leukemia treated with AALL0932, AALL0434, AALL1131, and AALL0631 for ALL and AAML0531 and AAML1031 for AML. We excluded patients diagnosed at an age younger than 2 years (*n*=7) (because BMI percentile calculation is not recommended for this age group [22], those who never achieved remission (*n*=3), patients with a secondary malignancy (*n*=2) and one patient with myelodysplastic syndrome. The final analysis included 181 patients (Figure 1). De-identified patient demographic and clinical information, including age, sex, insurance type, white blood cell (WBC) count, BMI at diagnosis, relapse, and mortality status as well as follow-up time were obtained from the data warehouse or through patient chart review.

BMI at diagnosis was calculated as weight (kilograms) divided by the square of height (meters). BMI percentiles were determined using the 2000 Centers for Disease Control and Prevention (CDC) BMI for age growth charts for children between 2 and 20 years of age [23]. Participants were classified according to age-adjusted BMI percentiles as overweight/obese (≥85^th^ percentile) and non-overweight (<85^th^ percentile). The insurance type was categorized as public (Medicaid, Medicaid HMO) versus private (all others) class.

### 2.2. Statistical Methods

Demographic and clinical characteristics were summarized by obesity status using median and range for continuous variables and counts and percentages for categorical variables. Duration of relapse-free survival (RFS) was defined as the time from diagnosis date to the first documentation of any relapse. Duration of overall survival (OS) was defined as the time from diagnosis date to the date of death. Patients who were lost to follow-up or who did not experience an event were censored at the date of last contact.

A complete set analysis and an analysis after multiple imputation of missing values for both response (relapse, *n*=28) and independent variables (BMI = 25, insurance type, *n*=5, and WBC, *n*=29) were performed. Five multiply imputed datasets were first generated. Each imputed dataset was analyzed using standard procedures, and then the results from the five datasets were combined for inference. Hazard ratios (HRs) and 95% confidence intervals (95% CI) from unadjusted and multivariable adjusted Cox proportional hazard models were used further to evaluate the association between RFS or OS and obesity on the imputed dataset. Multivariable models were adjusted for age (continuous), sex, and WBC (continuous) at diagnosis based on previous studies [24, 25] as well as insurance type (private/public), which is associated with disease outcome [26]. Log-transformed WBC was used in all models due to non-normality. In addition to analyses that combined all participants, subgroup analyses were conducted to investigate putative associations in different age groups. Previous studies have suggested that the associations might be different among children older than 10 years and those younger than 10 years [13, 17]. Therefore, subgroup analyses were conducted among children ≥10 years and children <10 years.

The number of eligible patients treated at our institution during the study period (*n*=181) determined the sample size. Assuming that 30% of the study participants were overweight/obese [27] and that relapse occurs in 20% of patients, the study had at least 80% power at the 5% significance level to detect a HR ≥ 2.8. Due to the small sample size of our study, we performed a meta-analysis using random effect models to pool our HRs from multivariable adjusted models with those of previous studies that were recently summarized by our group [28]. Statistical heterogeneity between study specific effects was evaluated with Cochran's Q test and quantified with I-squared statistic.

Descriptive statistics were performed using SAS version 9.4 and multiple imputation, Cox analyses and meta-analyses were performed using Stata v 15. All statistical tests were two-sided with a *p* value <0.05 considered statistically significant.

## 3. Results

Characteristics of patients with unimputed information on obesity status are shown in Table 1. Approximately 29% (45/156) of the patients were classified as overweight/obese. Compared to non-overweight patients, overweight/obese patients were older (median age: 13 vs 6 years), were likely to have public insurance (53.3% vs 44.1%), to be diagnosed with AML (24.4% vs 12.6%), had a higher median WBC count at diagnosis (11.9 × 10^3^ vs 7.5 × 10^3^/L) and a longer median follow-up time (61.7 months vs 45.4 months). Twenty-seven percent of overweight/obese developed relapse (compared to 19% non-overweight), and 29% of overweight/obese died (compared to 12% non-overweight). Death was mostly due to relapse (*n*=15), disease/treatment-related (*n*=7), or other (*n*=5). Results of unadjusted and multivariable-adjusted analyses were similar for imputed and complete set analyses (Tables 2 and 3). Age- and sex-adjusted Cox proportional hazard regression analyses (Table 2) did not show a statistically significant association between relapse and obesity status, although the association for children ≥10 years showed a trend towards increased risk of relapse for overweight/obese patients (imputed: HR = 2.89, 95% CI = 0.89–9.36, *p*=0.08; complete set: HR = 2.46, 95% CI = 0.96–7.27, *p*=0.06) that was not evident among children <10 years. However, the risks attenuated slightly and were not statistically significant after additional adjustment for insurance type and WBC at diagnosis (Supplementary Table 1). We observed a statistically significant association between mortality and obesity status in unadjusted models (imputed: HR = 2.54, 95% CI = 1.15–5.60, *p*=0.021; complete set: HR = 2.72, 95% CI = 1.26–5.91, *p*=0.011) that disappeared in both age- and sex-adjusted and multivariable-adjusted analyses (Table 3, Supplementary Table 1). Analyses limited to ALL, the most common type of leukemia, did not show an association between relapse or mortality (Table 4) and obesity status. As expected, analysis based on the small number of AML cases only did not show a statistically significant association for relapse (HR = 3.93, 95% CI = 0.71–21.82, *p*=0.12) or mortality (HR = 1.39, 95% CI = 0.31–6.27, *p*=0.67).

**Figure 2:**
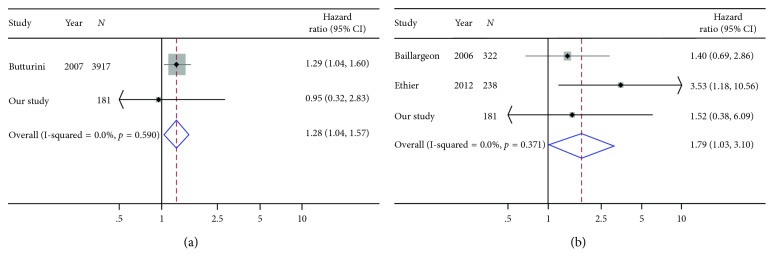
Meta-analyses combining the results of relapse (a) and overall survival (b) for ALL from this study with previous studies.

**Figure 3:**
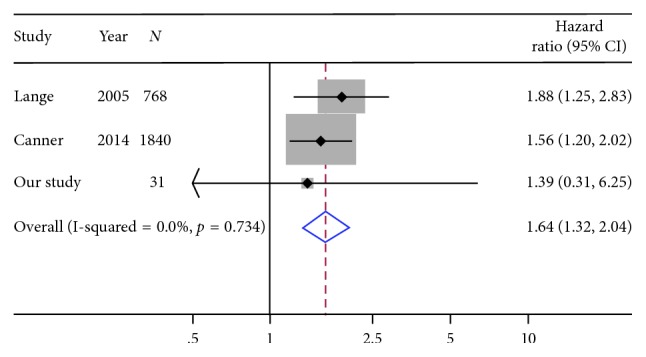
Meta-analyses combining the results of overall survival for AML from this study with previous studies.

A meta-analysis combining our HRs from the multivariable models with those of previous studies showed associations between obesity and increased risk for mortality (HR = 1.79, 95% CI = 1.03–3.10) and relapse (HR = 1.28, 95% CI = 1.04–1.57) for ALL, albeit the analysis for relapse included only two studies (Figure 2). Similarly, we observed an association between obesity and increased risk of mortality for AML (HR = 1.64, 95% CI = 1.32–2.04) (Figure 3). None of the studies that met inclusion for the meta-analysis reported an estimate for AML relapse.

## 4. Discussion

In the present study, we evaluated the association between obesity status and pediatric leukemia relapse and mortality. We observed a trend towards an increased risk of relapse associated with overweight/obesity in age- and sex-adjusted model among children ≥10 years, but the trend disappeared after additional adjustment for insurance type and WBC at diagnosis. We observed an association between overweight/obesity and mortality in unadjusted models, but the association disappeared in multivariable models that adjusted for factors including insurance type. However, the results of a meta-analysis that combined our findings with previous studies revealed an increased risk of mortality for overweight/obesity.

The relationship between obesity and cancer relapse is biologically plausible as obesity may affect molecular pathways that are relevant to cancer progression [29, 30]. Though this association has been observed in various adult cancers [10–12], findings on the association between obesity and pediatric leukemia relapse have been inconsistent. Weir et al. [20] examined the effects of standardized deviation score for BMI at diagnosis on leukemia relapse in children in the UK (*n*=1,025) and did not observe a statistically significant association between obesity and relapse. Another study in the UK on a cohort of 1,033 patients found no evidence to support the association between overweight/obesity at diagnosis and childhood leukemia relapse [16]. Findings from two similar studies in the US by Hijiya et al. [18] and Baillargeon et al. [17] also failed to detect an association between obesity at diagnosis and risk of relapse in children with ALL. Another study in Turkey did not show a difference between mean BMI at diagnosis between relapsed and nonrelapsed patients [19]. Our finding is consistent with these studies.

However, other studies have reported an increased risk of pediatric leukemia relapse with obesity. A large retrospective cohort study of 4,260 US patients with ALL found obesity at time of diagnosis to be an independent predictor of relapse in patients ≥10 years of age [13]. We observed a similar trend in our study, but the association disappeared after additional adjustment for insurance type and WBC at diagnosis. The lack of association in our multivariable analysis could potentially be due to the small sample size of our study. A smaller historical cohort study of 181 youths with ALL found an increased risk of relapse with obesity at diagnosis in patients <10 years of age [15]. Similarly in a previous smaller study (*n*=78), Reilly and colleagues observed an association between weight for height standard deviation score and risk for relapse, with the highest risk observed in children with a lower weight-for-height [14]. Among AML patients, Lohmann and colleagues did not find an association between BMI at diagnosis and prognosis for children aged 2–9 years, but observed a trend for improved outcome in overweight patients aged 10–17 years [31]. Inabi and colleagues observed a higher risk of mortality for overweight/obese AML patients [21]. Although our findings for AML were not statistically significant (due to small numbers), the observed HRs were in the direction of an increased risk. Future studies should investigate the association between obesity and AML outcomes.

Evidence from these previous studies suggest that the inconsistency in study findings may not be attributed to sample size, since an association or lack of an association is observed in both large and small studies. Our findings suggested a trend towards an increased risk of relapse for overweight/obese patients among children ≥10 years old. The association may be limited to a certain pediatric age group and thus not evident in studies that do not stratify on age. Therefore, future larger multicenter studies that will stratify analyses on age are warranted to provide further insights into the relationship between obesity at diagnosis and pediatric leukemia relapse. Existing evidence suggests that White Hispanics have a higher risk of relapse and mortality compared to other racial/ethnic groups [32, 33]. Since the distribution of obesity also varies by ethnicity [34], it is important to also evaluate the association between obesity and outcomes by race/ethnicity.

Our meta-analyses findings suggested an increased risk of mortality for overweight/obese patients that is consistent with the result of a recent meta-analysis [28]. The meta-analysis including a total of 916 cases from seven studies [17, 24, 35–39] showed an increased risk of mortality for overweight/obese patients and suggested that about 5% of the pediatric leukemia deaths could potentially be attributed to overweight/obesity. The management of obesity could therefore be considered during treatment to improve survival outcomes for patients with a high BMI at diagnosis.

Potential strengths of this study include the restriction of analyses to patients with leukemia (as some of the prior studies included patients with various forms of hematologic conditions [35, 36] and the adjustment for age, sex, WBC at diagnosis, and insurance type in our multivariable analyses. Information for BMI and WBC at diagnosis was missing for some patients because they were not diagnosed at our institution. We therefore performed multiple imputations for the missing information. The majority of previous studies performed a complete set analysis or/and did not adjust for insurance type (which is suggested to affect cancer outcome [40–43]) in multivariable models.

The main limitation of our study is the small sample size, which limited the analysis for each leukemia subtype. Lumping all forms of leukemias together could potentially distort associations with specific leukemias. We performed analyses limited to ALL, the most common type of leukemia. Though the effect estimates of these analyses did not vary appreciably from that of all leukemias combined, none of the associations were statistically significant possibly due to the small sample size. Another limitation is the grouping of overweight and obesity together. This grouping may represent a more heterogeneous adiposity profile, which could potentially mask an association between obesity and relapse or mortality. We could not also adjust for several important potential confounding factors such as cytogenetic-defined risk groups, T phenotype, B-precursor subgroup, immunophenotype, and minimal residual disease. In addition, the study was a secondary analysis of administrative data that were not collected for research purposes and more specifically, to investigate the relationship between obesity and pediatric leukemia relapse. As a result, we were limited in the scope of analysis that could be performed. We also used BMI as a measure of obesity. It is important to note that reliance on BMI as an anthropomorphic measure of obesity does not elucidate fat and lean muscle mass content. While BMI is a universally accepted method to approximate body composition, future studies would benefit from coupling it with other measures of body composition such as Dual energy X-ray absorptiometry (DEXA) scans and bioimpedance. Another limitation is the use of insurance type as a proxy for socioeconomic status (SES). We did not have information on parental educational level, occupation, and/or income, which may be better measures of SES. However, insurance status has been associated with income whereby public insurance is associated with low SES [44, 45]. In addition, we did not exclude patients with concomitant conditions that have been associated with obesity and leukemia outcomes such as Down syndrome. Despite these limitations, some of the trends observed in our study are interesting and could be evaluated further in larger studies.

Obesity represents a rare opportunity for preventive intervention that could improve outcomes; therefore, understanding the prognostic impact of obesity on pediatric cancer outcomes has both clinical and public health implications. Although our study did not find statistically significant associations between overweight/obesity and relapse or mortality due to the small sample size, the findings from meta-analyses revealed an increased risk of mortality for overweight/obese patients. In addition, our findings may suggest a potential association between obesity and relapse that may be limited to children ≥10 years. However, our study was based on a small sample size from a single institution, and this association needs to be further investigated in larger, multicenter studies.

## Figures and Tables

**Table 1:** Characteristics at diagnosis for pediatric leukemia patients by obesity status.

	All		Obesity			
			Non-overweight (*n*=111)		Overweight/obese (*n*=45)	
	*N*	%	*N*	%	*N*	%
Gender						
Female	73	46.79	51	45.95	22	48.89
Male	83	53.21	60	54.05	23	51.11
Age at diagnosis (yrs), median (range)	156	7 (2–20)	111	6 (2–19)	45	13 (2–20)
<10	102	65.38	84	75.68	18	40
≥10	54	34.62	27	24.32	27	60
Race/ethnicity						
Asian	6	3.85	4	3.6	2	4.44
Black	11	7.05	7	6.31	4	8.89
Caucasian	98	62.82	76	68.47	22	48.89
White Hispanic	34	21.79	19	17.12	15	33.33
Other	7	4.49	5	4.5	2	4.44
Insurance type						
Private	80	51.28	62	55.86	18	40
Public	73	46.79	49	44.14	24	53.33
Missing	3	1.92	0	0	3	6.67
Type of leukemia						
ALL	123	78.85	93	83.78	30	66.67
AML	25	16.03	14	12.61	11	24.44
^*∗*^Other	8	5.13	4	3.6	4	8.89
WBC, median (range)	9.46	(0.21–744.0)	7.45	(0.72–402.6)	11.86	(0.21–744.0)
<50 × 10^3^/L	118	75.64	87	78.38	31	68.89
≥50 × 10^3^/L	25	16.03	13	11.71	12	26.67
Missing	13	8.33	11	9.91	2	4.44
Follow-up time (months), median (range)	57.04	(0.42–260.75)	45.42	(0.92–250.5)	61.67	(0.42–260.75)

^*∗*^Other includes Burkitt cell leukemia, chronic myelogenous leukemia, BCR/ABL positive, chronic myeloid leukemia, NOS, anaplastic large cell lymphoma, T-cell and Null-cell type, and juvenile myelomonocytic leukemia.

**Table 2:** Hazard ratios for the association between obesity status and relapse-free survival.

	Imputed				Complete set			
Variables	HR^*∗*^	Lower 95% CI	Upper 95% CI	*p*-value	HR^*∗*^	Lower 95% CI	Upper 95% CI	*p*-value
Unadjusted								
All								
Overweight/obese vs non-overweight	1.77	0.91	3.46	0.09	1.75	0.85	3.40	0.13
<10 years								
Overweight/obese vs non-overweight	0.52	0.08	3.39	0.48	0.33	0.04	2.56	0.29
≥10 years								
Overweight/obese vs non-overweight	2.39	0.79	7.26	0.12	2.17	0.78	6.01	0.14

Sex and/or age adjusted								
All								
Overweight/obese vs non-overweight	1.57	0.71	3.45	0.26	1.45	0.67	3.14	0.34
Female vs male	0.56	0.29	1.08	0.08	0.46	0.22	0.99	0.046
Age at diagnosis	1.07	1.02	1.13	0.012	1.11	1.04	1.18	0.002
<10 years								
Overweight/obese vs non-overweight	0.52	0.08	3.54	0.49	0.31	0.04	2.46	0.27
Female vs male	1.01	0.42	2.43	0.99	1.23	0.44	3.46	0.70
≥10 years								
Overweight/obese vs non-.overweight	2.89	0.89	9.36	0.08	2.46	0.96	7.27	0.06
Female vs male	0.31	0.11	0.90	0.031	0.19	0.05	0.67	0.009

^*∗*^Hazard ratio.

**Table 3:** Hazard ratios for the association between obesity status and overall survival.

	Imputed				Complete set			
Variables	HR^*∗*^	Lower 95% CI	Upper 95% CI	*p*-value	HR^*∗*^	Lower 95% CI	Upper 95% CI	*p*-value
Unadjusted								
All								
Overweight/obese vs non-overweight	2.54	1.15	5.60	0.021	2.72	1.26	5.91	0.011
<10 years								
Overweight/obese vs non-overweight	0.93	0.10	8.57	0.94	1.07	0.12	9.59	0.95
≥10 years								
Overweight/obese vs non-overweight	1.74	0.67	4.56	0.25	1.75	0.70	4.36	0.23

Sex and/or age adjusted								
All								
Overweight/obese vs non-overweight	1.60	0.62	4.12	0.32	1.72	0.75	3.93	0.20
Female vs male	0.48	0.22	1.08	0.08	0.40	0.16	0.95	0.039
Age at diagnosis	1.18	1.09	1.27	<0.001	1.20	1.10	1.30	<0.001
<10 years								
Overweight/obese vs non-overweight	0.89	0.09	8.62	0.92	1.05	0.17	9.39	0.97
Female vs male	1.63	0.36	7.32	0.53	1.61	0.27	9.65	0.60
≥10 years								
Overweight/obese vs non-overweight	2.13	0.77	5.88	0.14	2.22	0.89	5.51	0.09
Female vs male	0.29	0.10	0.80	0.017	0.22	0.07	0.66	0.007

^*∗*^Hazard ratio.

**Table 4:** Association between obesity and relapse or mortality among pediatric ALL patients.

	Imputed				Complete set			
Variables	HR^*∗*^	Lower 95% CI	Upper 95% CI	*p*-value	HR^*∗*^	Lower 95% CI	Upper 95% CI	*p*-value
Relapse								
Unadjusted								
Overweight/obese vs non-overweight	1.09	0.43	2.76	0.86	0.99	0.36	2.67	0.98

Sex and age adjusted								
Overweight/obese vs non-overweight	1.00	0.35	2.84	0.99	0.88	0.31	2.48	0.81
Female vs male	0.79	0.37	1.69	0.55	0.65	0.27	1.55	0.33
Age at diagnosis	1.05	0.98	1.12	0.17	1.09	1.00	1.17	0.04

Multivariable adjusted								
Overweight/obese vs non-overweight	0.95	0.32	2.84	0.93	0.74	0.22	2.46	0.62
Female vs male	0.71	0.33	1.53	0.38	0.65	0.25	1.68	0.37
Age at diagnosis	1.07	0.99	1.16	0.08	1.13	1.03	1.25	0.011
Public vs private	1.87	0.80	4.38	0.15	2.20	0.79	6.15	0.13
WBC at diagnosis	0.97	0.73	1.28	0.81	0.92	0.68	1.26	0.61

Mortality								
Unadjusted								
Overweight/obese vs non-overweight	2.58	0.84	7.92	0.10	2.83	0.95	8.46	0.06
Sex and age adjusted								
Overweight/obese vs non-overweight	1.61	0.43	6.33	0.47	1.89	0.60	6.00	0.28
Female vs male	0.77	0.27	2.20	0.63	0.52	0.16	1.74	0.29
Age at diagnosis	1.20	1.09	1.33	<0.001	1.25	1.10	1.42	<0.001
Multivariable adjusted								
Overweight/obese vs non-overweight	1.52	0.38	6.10	0.54	2.00	0.50	8.12	0.33
Female vs male	0.63	0.21	1.88	0.41	0.35	0.08	1.46	0.15
Age at diagnosis	1.21	1.09	1.34	<0.001	1.30	1.12	1.52	0.001
Government vs commercial	2.24	0.70	7.13	0.17	1.98	0.51	7.62	0.32
WBC at diagnosis	1.24	0.93	1.64	0.14	1.21	0.87	1.68	0.26

^*∗*^Hazard ratio.

## Supplementary Materials

Supplementary Materials Supplementary Table 1: multivariable adjusted models for the association between obesity and relapse or mortality among pediatric leukemia patients.
