# Supplementary material for: Body Mass Index at Pediatric Leukemia Diagnosis and the Risks of Relapse and Mortality: Findings from a Single Institution and Meta-analysis
Source: J Obes. 2018 Nov 1;2018:7048078. doi: 10.1155/2018/7048078 (PMC6236800; doi:10.1155/2018/7048078)
Supplement: Supplementary Materials — Supplementary Table 1: multivariable adjusted models for the association between obesity and relapse or mortality among pediatric leukemia patients. [file 7048078.f1.docx]

| Supplementary Table 1. Multivariate adjusted models for the association between obesity and relapse or mortality among pediatric leukemia patients | | | | | | | | |
| --- | --- | --- | --- | --- | --- | --- | --- | --- |
|  | Imputed | | | | Complete set | | | |
| Variables | HR* | Lower 95%CI | Upper 95%CI | P-value | HR* | Lower 95%CI | Upper 95%CI | P-value |
| **Relapse** | | | | | | | | |
| *All* |  |  |  |  |  |  |  |  |
| Overweight/obese vs non-overweight | 1.42 | 0.61 | 3.31 | 0.41 | 1.16 | 0.46 | 2.95 | 0.76 |
| Female vs Male | 0.51 | 0.27 | 1.00 | 0.049 | 0.53 | 0.23 | 1.20 | 0.13 |
| Age at diagnosis | 1.09 | 1.02 | 1.15 | 0.006 | 1.13 | 1.04 | 1.22 | 0.002 |
| Public vs Private | 1.77 | 0.89 | 3.52 | 0.10 | 1.85 | 0.81 | 4.23 | 0.14 |
| WBC at diagnosis | 0.96 | 0.77 | 1.21 | 0.75 | 0.92 | 0.73 | 1.17 | 0.50 |
| *< 10 years* |  |  |  |  |  |  |  |  |
| Overweight/obese vs non-overweight | 0.48 | 0.07 | 3.11 | 0.44 | 0.38 | 0.04 | 3.14 | 0.36 |
| Female vs Male | 0.98 | 0.40 | 2.43 | 0.97 | 1.41 | 0.42 | 4.734 | 0.58 |
| Public vs Private | 1.12 | 0.46 | 2.75 | 0.80 | 1.41 | 0.40 | 4.99 | 0.59 |
| WBC at diagnosis | 1.16 | 0.74 | 1.82 | 0.51 | 1.03 | 0.57 | 1.87 | 0.91 |
| ≥ *10 years* |  |  |  |  |  |  |  |  |
| Overweight/obese vs non-overweight | 2.26 | 0.60 | 8.45 | 0.22 | 1.62 | 0.46 | 5.67 | 0.45 |
| Female vs Male | 0.30 | 0.10 | 0.87 | 0.026 | 0.27 | 0.07 | 0.98 | 0.047 |
| Public vs Private | 2.07 | 0.67 | 6.44 | 0.21 | 1.59 | 0.46 | 5.50 | 0.46 |
| WBC at diagnosis | 0.92 | 0.71 | 1.20 | 0.54 | 0.89 | 0.67 | 1.18 | 0.42 |
| **Mortality** | | | | | | | | |
| **Multivariable adjusted** |  |  |  |  |  |  |  |  |
| *All* |  |  |  |  |  |  |  |  |
| Overweight/obese vs non-overweight | 1.39 | 0.55 | 3.54 | 0.10 | 1.55 | 0.54 | 4.44 | 0.41 |
| Female vs Male | 0.46 | 0.20 | 1.03 | 0.06 | 0.27 | 0.09 | 0.84 | 0.023 |
| Age at diagnosis | 1.18 | 1.089 | 1.28 | <0.001 | 1.27 | 1.13 | 1.43 | <0.001 |
| Public vs Private | 2.27 | 0.97 | 5.33 | 0.06 | 2.01 | 0.74 | 5.48 | 0.17 |
| WBC at diagnosis | 1.21 | 0.98 | 1.51 | 0.08 | 1.13 | 0.89 | 1.44 | 0.31 |
| *< 10 years* |  |  |  |  |  |  |  |  |
| Overweight/obese vs non-overweight | 0.45 | 0.04 | 5.28 | 0.52 | 2.76 | 0.14 | 53.45 | 0.50 |
| Female vs Male | 1.53 | 0.26 | 9.10 | 0.64 | - | - | - | - |
| Public vs Private | 1.87 | 0.29 | 12.22 | 0.51 | 1.65 | 0.08 | 32.89 | 0.74 |
| WBC at diagnosis | 2.24 | 1.14 | 4.42 | 0.021 | 1.95 | 0.67 | 5.63 | 0.22 |
| ≥ *10 years* |  |  |  |  |  |  |  |  |
| Overweight/obese vs non-overweight | 1.62 | 0.58 | 4.54 | 0.36 | 1.35 | 0.45 | 4.04 | 0.59 |
| Female vs Male | 0.29 | 0.10 | 0.80 | 0.017 | 0.30 | 0.10 | 0.95 | 0.041 |
| Public vs Private | 3.48 | 1.08 | 11.19 | 0.036 | 3.09 | 0.96 | 9.96 | 0.058 |
| WBC at diagnosis | 1.04 | 0.83 | 1.31 | 0.71 | 1.03 | 0.81 | 1.32 | 0.80 |
